# The Story of the Insane from Year to Year

**Published:** 1904-02-13

**Authors:** 


### THE STORY OF THE INSANE FROM YEAR TO YEAR.

SIXTEENTH ANNUAL SERIES.

### THE ROYAL ASYLUM, ABERDEEN.

On the first of January 929 patients were in residence, and at the end of the year there were 958. The first admissions were 232, the re-admissions 72, the recoveries 140, and the deaths 81. The average number resident during the year was 928, and the total number under treatment was 1,215. The recoveries, calculated on the admissions, stand at 46 per cent.; and the deaths, calculated on the average number resident, at 8·7. Of the deaths 32 were caused by cerebral and spinal diseases, 8 by pneumonia, and 6 by consumption. In nine of the year's admissions the insanity was due to domestic trouble, adverse circumstances, and religion; in 76 to hereditary influence; in 45 to previous attacks; and in 29 to intemperance in drink. Assuredly nothing so much conduces to recovery from insanity as does early treatment, and assuredly also medical superintendents often enough point out that fact in their annual reports; but it is to be feared that their words do not reach the people who might be influenced by them. We, therefore, gladly give a little more publicity to the following sentences of Dr. Reid:—"It is a pity that the general public are so slow to believe that mental affections are in their causes and means of cure similar to physical, and that early attention, with proper medical treatment, is essential to the chances of recovery. Private patients are too often retained to be subjected to every conceivable obsolete mode of treatment; and incurability is stamped on the face of them before they are removed from their relatives to be under the requisite care and supervision." The main asylum is overcrowded, and yet the death-rate from consumption is low, and it would further seem that the great majority of patients who died of the disease brought it to the asylum with them. In fact, there was not a single active case of tubercular disease present on the date of the report; and Dr. Reid attributes this immunity, no doubt correctly, to the amount of outdoor exercise enjoyed by the patients. "In nearly all cases they are out from seven in the morning till sunset in winter, and till eight o'clock in summer, and this in all weathers." This is a lesson to English medical superintendents, for certainly if such a regulation can be carried out in the colder north-east of Scotland it could much easier have effect given to it in our warmer English climate. But, one is inclined to ask, how is the work of the asylum carried on, and is not steady employment one of the most efficient means of cure?

### ROYAL ASYLUM, MONTROSE.

THIS asylum contained 679 patients at the beginning of the year, and 687 at the end of it. There were 133 first admissions, 28 readmissions, 63 recoveries, 11 discharged relieved, and 77 deaths. The average number resident during the year was 690, and the total number under treatment was 840. The recoveries were 39·12 per cent. of the admissions, and the deaths were 12·22 per cent. of the average number resident. Of the year's deaths 31 were caused by cerebral and spinal diseases, and 14 by consumption. Seven of the cases admitted owed their insanity to moral causes, 26 to hereditary influence, 19 to intemperance in drink, 15 to old age, and in 57 no cause was known. The table is therefore not of much use for purposes of comparison with other asylums. A villa for the reception of female patients is being built, the agreements with the District Lunacy Boards have been re-arranged, and Dr. Havelock is able to convey the pleasing intelligence that the population of the Montrose Asylum has now reached its high-water mark. Dr. Havelock reviews the position of the asylum in relation to the segregation system of treating consumption, and he finds that the death-rate from phthisis for five years amounted to 15 per thousand resident, and that this proportion is about 5 per thousand lower than in the public asylums of the kingdom. He further finds that of the 14 deaths from consumption which took place last year, 9 of the patients were admitted with the disease certainly diagnosed; 3 were suspected of having it, and in the remaining 2 it was developed after long residence in the asylum. It is Dr. Havelock's rule to treat his phthisical cases in separate wards, and he thinks it is wisest to follow this plan, "until experience has been gained in the working of these sanatoria" attached to our county asylums. There were 121 private patients in residence, and nearly £6,000 was received for their maintenance. The remaining 570 were paupers chargeable to parishes in Forfarshire, Dundee, Kincardineshire, Caithness, Shetland, and Orkney. Private patients pay from £25 to £105 per annum, and a few are received at much higher rates.

### THE WORCESTER COUNTY ASYLUM.

HERE the numbers were 1,180 on the first of January and 1,158 on the 31st of December. There were 228 first-admissions, 38 readmissions, 96 recoveries, 15 discharged relieved, 61 discharged not improved, and 116 deaths. The average number resident during the year was 1,189, and the total number under treatment was 1,438. The recoveries were 39·5 per cent. of the admissions, and the deaths were 9·7 per cent. of the average number resident. Of the year's deaths 29 were caused by cerebral and spinal diseases, 19 by consumption, 9 by pneumonia, 16 by colitis, and 1 by enteric fever. Twenty-five of the year's admissions owed their insanity to moral causes, 74 to hereditary influence and family predisposition, and only 13 to drink. The asylum was overcrowded, and 45 patients were boarded out at the Derby and Northampton Asylums. Mechanical restraint was used in the case of one patient to prevent self-injury, and seclusion has been occasionally employed. One of the charge nurses was superannuated during the year, and received a pension of £26 a year. It is 50 years since the Worcester County Asylum was opened, and its growth and the increase in the number of patients under treatment are typical of most of our public asylums. In the year 1853 it contained 178 patients. In 1856 additions for 110 patients were put up, and this sufficed for five years, when another 45 beds were added. In 1863 wards for another 100 patients were built. At that time the medical superintendent occupied rooms in the main building; but in 1867 a detached house was provided for him, and the old rooms were fitted up for 54 patients. In 1871 further accommodation was called for, and new blocks were erected for 134 men. These had to be followed in 1885 by new wards for 210 patients, and in 1893 another 140 beds were put on. Finally, in 1898 the accommodation was again increased, this time for 140 female patients. During all this time the committee and their medical officers have done everything that could be suggested for the welfare and for the cure of their patients, and the average recovery rate has been almost 35 per cent. of the annual admissions. But if we were to ask what has been done to stem this torrent of disease by attempting to prevent its recurrence, we should probably get no answer. Insanity has not yet taken its place in preventive medicine. The committee express their "appreciation of the manner in which Mr. Braine-Hartnell and the assistant medical officers have continued to discharge the very responsible duties which devolve upon them." The weekly cost of maintenance per head is a fraction under 8s.

